# Antimicrobial Activity of *Acanthus ilicifolius* (L.)

**DOI:** 10.4103/0250-474X.49134

**Published:** 2008

**Authors:** S. Bose, Arti Bose

**Affiliations:** Department of Pharmacognosy, Gupta College of Technological Sciences, College of Pharmacy Ashram More, G. T. Road, Asansol-713 301, India

**Keywords:** *Acanthus ilicifolius*, agar cup-plate method, antimicrobial activity, mangrove

## Abstract

The antimicrobial activity of alcoholic, butanolic and chloroform extracts of leaves and roots of the plant *Acanthus ilicifolius* ware studied. Ampicillin and clotrimazole were used as standard antibacterial and antifungal agents respectively. The result of the study revealed that the alcoholic extract and chloroform extract of leaves exhibited strong inhibitory action against *Bacillus subtilis, Staphylococcus aureus, Candida albicans, Aspergillus fumigatus* and *Aspergillus niger* and moderate inhibitory action against *Pseudomonas aeruginosa* and *Proteus vulgaris*. The rest of the extracts showed moderate activity.

*Acanthus ilicifolius* is a spiny herb found in mangrove of southern Thailand. In folklore and traditional practice, different parts of *A. ilicifolius* (Acanthaceae) have been used to treat rheumatism, asthma, paralysis, psoriasis and leucorrhoea^1^. Antioxidant, hepatoprotective, leishmanicidal, tumour reducing and anticancer activities of various extracts of *A. ilicifolius* have been reported^2^–^4^. These created an interest to test the possible antimicrobial activity of different parts of this plant, which has not been reported; hence, the present study was designed. The phytochemical literature reveals the presence of 2-benzoxazolinone, lignan glucosides, benzoxazinoide glucosides, flavone glycosides and phenylethanoid glycosides in this plant^5^–^8^. The present study was aimed at the preliminary investigation of antibacterial and antifungal activity of alcoholic, butanolic and chloroform extracts of leaves and roots of *A. ilicifolius*.

Leaves and roots of *A. ilicifolius* were collected from Netravati river valley of Mangalore in the month of October. The plant was authenticated in the Department of Botany, Netaji Mahavidyalaya, District Hoogly, West Bengal. The collected plant materials were shade dried at room temperature and mechanically reduced separately to course powders. The powders of leaves and roots (100 g each) were then extracted individually with 95% alcohol, butanol and chloroform in a Soxhlet apparatus by continuous heat extraction for 78 h. The extracts so obtained were concentrated to dryness by evaporating the solvents under reduced pressure.

The *in vitro* antibacterial and antifungal studies of the ethanolic, butanolic and chloroform extracts of the leaves and roots were carried out by the Agar cup-plate method^9^. All the extracts were separately dissolved in dimethylsulfoxide (DMSO) to get 10 mg/ml solutions. Ampicillin (1 mg/ml) and clotrimazole (1 mg/ml) were used as standard antibacterial and antifungal agents respectively. The antibacterial activity was evaluated by employing 24 h cultures of *Bacillus subtilis*, *Staphylococcus aureus*, *Pseudomonas aeruginosa* and *Proteus vulgaris* using Muller Hinton Agar medium. Antifungal activity was carried out against 24 h cultures of *Candida albicans*, *Aspergillus fumigatus* and *Aspergillus niger* using Sabouraud dextrose agar medium. The bacterial and fungal strains employed in the study were obtained from NCIM, Pune. Accurately 0.2 ml of the test and standard solutions were transferred to cups aseptically and labeled accordingly. The microorganism inoculated plates were then maintained at room temperature for 2 h to allow the diffusion of the solutions into the medium. The petri dishes used for antibacterial screening were incubated at 37±1° for 24 h, while those used for antifungal activity were incubated at 28±1° for 48 h. The diameters of zone of inhibition surrounding each of the wells were recorded.

Table 1 enumerates the antibacterial and antifungal activity of the extracts of different parts of the title plant. The ethanol, butanol and chloroform extracts of the different parts of the plant exhibited strong to moderate activity against the test microorganisms. The results revealed that, the alcoholic and chloroform extracts of leaves exhibited strong inhibitory action against *Bacillus subtilis*, *Staphylococcus aureus*, *Candida albicans*, *Aspergillus fumigatus* and *Aspergillus niger* and moderate inhibitory action against *Pseudomonas aeruginosa* and *Proteus vulgaris*. The rest of the extracts showed moderate activity.

**TABLE 1:** ANTIMICROBIAL ACTIVITY OF *ACANTHUS ILICIFOLIUS* (L.)

Micro Organisms	Standards (mm)	Zone of inhibition (mm)					
		
		Alcohol Extract (10 mg/ml)		Butanol Extract (10 mg/ml)		Chloroform Extract (10 mg/ml)	
				
		Leaves	Roots	Leaves	Roots	Leaves	Roots
*B. subtilis*	25a	20	17	16	14	22	16
*S. aureus*	24a	18	16	08	12	21	11
*P. aeruginosa*	20a	18	14	10	10	20	13
*P. vulgaris*	21a	20	14	12	15	19	15
*C. albicans*	26b	22	20	15	14	26	20
*A. fumigatus*	28b	19	18	19	12	22	20
*A. niger*	28b	21	20	12	12	20	19

a is ampicillin and b is clotrimazole. Each zone of inhibition is an average of three independent determinations and the solvent (DMSO) did not show any inhibition.

## References

[1] Kirtikar KR, Basu BD (2001). Indian Medicinal Plants.

[2] Babu BH, Shylesh BS, Padikkala J (2001). Antioxidant and hepatoprotective effect of *Acanthus ilicifolius*. Fitoterapia.

[3] De Carvalho PB, Ferreira EI (2001). Leishmaniasis phytotherapy: Nature's leadership against an ancient disease. Fitoterapia.

[4] Babu BH, Shylesh BS, Padikkala J (2002). Tumour reducing and anticarcinogenic activity of *Acanthus ilicifolius*. J Ethnopharmacol.

[5] Kanchanapoom T, Kamel MS, Kasai R, Yamasaki K, Picheansoonthon C, Hiraga Y (2001). Lignan glucosides from *Acanthus ilicifolius*. Phytochemistry.

[6] Kanchanapoom T, Kamel MS, Kasai R, Yamasaki K, Picheansoonthon C, Hiraga Y (2001). Benzoxazinoid glucoside from *Acanthus ilicifolius*. Phytochemistry.

[7] Wu J, Zhang S, Xiao Q, Li Q, Huang J, Long L, Huang L (2003). Megastigmane and flavone glycosides from *Acanthus ilicifolius*. Pharmazie.

[8] Wu J, Huang J, Xiao Q, Zhang S, Xiao Z, Li Q (2004). Complete assignments of ^1^H and ^13^C NMR data for 10 phenylethanoid glycosides. Magn Reson Chem.

[9] Barry AL (1976). The antimicrobial susceptibility test principle and practices.

